# Associations between smoking status and bodily pain in a cross-sectional survey of UK respondents

**DOI:** 10.1016/j.addbeh.2019.106229

**Published:** 2020-03

**Authors:** Olga Perski, Claire Garnett, Lion Shahab, Jamie Brown, Robert West

**Affiliations:** a Research Department of Behavioural Science and Health, University College London, 1-19 Torrington Place, London WC1E 6BT, UK

**Keywords:** Tobacco smoker, Former daily smoker, Pain, Cross-sectional, BBC Lab UK Study

## Abstract

**Background:**

Research indicates that ex- and current smokers report increased levels of bodily pain compared with never smokers. This could be secondary to smoking-related disease or psychological characteristics of smokers, or it could be a neurological or vascular effect of a period of regular smoking.

**Aims:**

We compared self-reported levels of bodily pain in daily, never daily and former daily smokers stratified by age group and adjusting for a wider range of covariates than has been undertaken to-date, including health status, neuroticism, anxiety and depression.

**Method:**

223,537 UK respondents aged 16+ years were surveyed between 2009 and 2013 in the British Broadcasting Corporation (BBC) Lab UK Study. Respondents provided information on bodily pain, smoking status and a range of sociodemographic, health, behavioural and psychological characteristics.

**Results:**

After adjusting for all covariates, in 16-34-year-olds, reported levels of bodily pain in former daily smokers (B_adj_ = 0.72, 95% CI = 0.30, 1.15, *p* < .001) and daily smokers (B_adj_ = 0.50, 95% CI = 0.18, 0.82, *p* < .01) were higher than in never daily smokers. Reported levels of bodily pain were also higher in former daily smokers than in never daily smokers in those aged 35–64 (B_adj_ = 1.04, 95% CI = 0.69, 1.38, *p* < .001) and 65 + years (B_adj_ = 1.65, 95% CI = 0.07, 3.24, *p* < .05).

**Conclusions:**

After adjusting for key characteristics, former daily smokers reported higher levels of bodily pain compared with never daily smokers at all ages. This raises the possibility that a period of smoking may have lasting effects on pain experiences.

## Highlights

• Former daily smokers reported higher levels of bodily pain compared with never daily smokers at all ages.
• In 16-34-year-olds, daily smokers reported higher levels of pain compared with never daily smokers.
• A period of regular smoking may affect pain experiences.

## 1 Introduction

Cigarette smoking remains one of the leading causes of premature morbidity and mortality with 7 million people worldwide dying annually of a smoking-related disease (World Health Organization, 2017). Pain is also a global public health problem; it has been estimated that 20% of adults suffer from acute, chronic or intermittent pain, or a combination of all three (Goldberg & McGee, 2011). Pain is defined as “a distressing experience associated with actual or potential tissue damage with sensory, emotional, cognitive and social components” (Williams & Craig, 2016). Pain is implicated in absenteeism and reduced levels of productivity (Phillips, 2009), resulting in large costs to society. Research has found that ex- and current smokers report increased levels of bodily pain relative to never smokers (Shi et al., 2010, Ditre et al., 2011, Bastian et al., 2015, Jakobsson, 2008, John et al., 2006). Compared with lifetime non-smokers, occasional and regular lifetime smokers in the 1946 British birth cohort study had an increased risk of reporting chronic widespread pain at the age of 68 years (Bendayan, Cooper, & Muthuri, 2018). This raises the possibility that a period of smoking at any time during the lifespan results in increased pain. However, it is also possible that the association is attributable to common causes or that it is accounted for by specific smoking-related diseases. To gain a better understanding of this issue, we compared bodily pain reported by former daily and daily smokers with never daily smokers in a large, cross-sectional sample of respondents from the British Broadcasting Corporation (BBC) Lab UK Study after adjusting for a wider range of covariates than has been undertaken to-date. We also stratified the analyses by age.

Although nicotine has acute analgesic effects (Shi, Weingarten, Mantilla, Hooten, & Warner, 2010), it is only one of ~5000 constituents of cigarette smoke (Bernhard & Wick, 2006). Research suggests that smoking is implicated in the development of pain even after relatively short periods of time; for example, a prospective cohort study of adolescents in Finland found that smoking at the age of 16 years was associated with an increased risk of reporting lower back pain at 18 years (Mikkonen et al., 2008). There is some evidence that quitting smoking does not lead to pain reduction (Bastian et al., 2015, Jakobsson, 2008, John et al., 2006), although it has also been found that activity in cortico-striatal circuits which have been implicated in the transition from acute to chronic pain decreases following smoking cessation (Petre et al., 2015) and that those who quit smoking during pain treatment have better outcomes (Behrend et al., 2012).

There are several plausible explanations for these findings. First, smoking may lead to chronic downregulation of the hypothalamic–pituitaryadrenal (HPA) axis. Hence, the analgesic effect of HPA axis activation which typically occurs during exposure to social or physiological stressors may be attenuated in smokers (Parkerson, Zvolensky, & Asmundson, 2013). Secondly, smoking may damage bones, joints and ligaments through vasoconstriction or hypoxia (Palmer, Syddall, Cooper, & Coggon, 2003). Thirdly, people who take up smoking may differ systematically from those who do not with regards to personality traits (e.g. neuroticism) or illness representations (e.g. the tendency to experience psychological distress as somatic symptoms) which are also associated with the development of pain (Palmer et al., 2003). For example, smokers are more likely than non-smokers to report increased levels of neuroticism (Munafò, Zetteler, & Clark, 2007), and neuroticism is commonly reported amongst individuals with chronic pain (Dersh, Polatin, & Gatchel, 2002).

Sociodemographic, behavioural and health-related factors that may be implicated in the smoking-pain relationship include sex, age, income level, self-rated health status, symptoms of anxiety and depression and alcohol consumption. Sex has previously been found to moderate the smoking-pain relationship, with female smokers reporting higher levels of pain than male smokers (Jakobsson and Larsson, 2014, John et al., 2006, Mikkonen et al., 2008). Older age may serve as a proxy for length or pack-years of smoking in current smokers and time since quitting in ex-smokers, and income level may serve as a proxy for social position. Prior evidence suggests that pack-years of smoking is positively associated with pain severity in daily smokers without chronic pain (De Vita, Maisto, Ansell, Zale, & Ditre, 2019). Although there is yet no conclusive evidence as to whether smoking exposure (e.g. length, intensity) is associated with greater odds of developing bodily pain over time, a tentative signal that increased pain in ex- and current smokers is caused by a period of smoking would be if it were found at all ages in a cross-sectional sample. This is because younger ex- and current smokers would have had only a relatively short period of smoking and would not have had time to develop major smoking-related diseases, so there would be little confounding with other factors likely to increase pain. Moreover, global ratings of self-rated health status or physical functioning are negatively associated with pain ratings (Jenkinson, Coulter, & Wright, 1993). Researchers have also examined the potentially mediating role of anxiety and depression on smoking in those with chronic pain (Zale, Maisto, & Ditre, 2016). Pain is positively associated with negative affect, which smoking can help alleviate (Parkerson et al., 2013). Experimental studies have highlighted the acute analgesic effect of ethanol (James, Duthie, Duffy, McKeag, & Rice, 1978), and some of those experiencing chronic pain use alcohol as a pain relief (Dersh et al., 2002, Radwanski, 1992, Brennan et al., 2005).

We therefore used data from the BBC Lab UK Study to answer the following questions:

1. Do daily and former daily smokers experience greater levels of bodily pain compared with never daily smokers after adjusting for age, sex, income level, self-rated health status, symptoms of anxiety and depression, neuroticism and frequency of binge drinking?
2. Is any increase in pain similar in younger and older respondents?

## 2 Methods

### 2.1 Study design and setting

The study protocol and analysis plan were pre-registered on the Open Science Framework (osf.io/kaud9). This was a correlational study involving cross-sectional data. The STROBE guidelines were used in the design and reporting of this study (Von Elm et al., 2007). A series of open access online surveys were hosted by the BBC Lab UK Study website between 2009 and 2013 (British Broadcasting Corporation, 2014). Anyone able to access the website could take part.

### 2.2 Inclusion criteria

Respondents were included if they were aged 16+ years and resided in the UK.

### 2.3 Sample recruitment

Interested respondents were invited to take part in open access experiments and surveys via the BBC Lab UK website (British Broadcasting Corporation, 2014). The survey was advertised and promoted via various BBC websites, radio programmes and television shows. This was a citizen science project, with data being collected by members of the general public in collaboration with scientists. As such, participants were not reimbursed for their time.

### 2.4 Ethical approval

The authors assert that all procedures contributing to this work comply with the ethical standards of the relevant national and institutional committees on human experimentation and with the Helsinki Declaration of 1975, as revised in 2008. This study involved secondary analyses of fully anonymised data obtained from the BBC Lab UK Study. Hence, specific ethical approval was not sought. Respondents were told that by clicking on the link to proceed to the survey, they were giving their consent to participate. Initiating the survey was used as a record of consent.

### 2.5 Measures

#### 2.5.1 Outcome variable

The outcome variable was the level of bodily pain in the past 4 weeks, assessed by two items from the validated Short Form-36 (SF-36) Health Survey: “How much bodily pain have you had during the past 4 weeks?” and “During the past 4 weeks, how much did pain interfere with your normal work (including both work outside the home and housework)?”. Response options for item 1 were: 1) none; 2) very mild; 3) mild; 4) moderate; 5) severe; and 6) very severe. Response options for item 2 were: 1) not at all; 2) slightly; 3) moderately; 4) quite a bit; 5) extremely. As per scoring instructions for the SF-36 Health Survey, item scores were summed and transformed into a scale ranging from 0 to 100 using the following formula: 100*(actual raw score-lowest possible raw score)/possible raw score range. To aid interpretation, reverse scoring was applied subsequently such that higher scores indicated greater levels of bodily pain. As a sensitivity analysis, responses to item 1 were also dichotomised into ‘no pain’ (response option 1) and ‘some pain’ (response options 2–6).

#### 2.5.2 Explanatory variable

The explanatory variable was smoking status, assessed by combining responses to the following two items: “Have you ever smoked cigarettes daily, that is, at least one cigarette every day for 30 days?” and “During the past 30 days, on average how many cigarettes did you smoke per day?”. Respondents indicating that they had never smoked cigarettes daily were coded as a ‘never daily smoker’, with those indicating that they had smoked cigarettes daily but 0 cigarettes in the past 30 days coded as a ‘former daily smoker’. Those indicating that they had smoked cigarettes daily and any cigarettes in the past 30 days were coded as a ‘daily smoker’.

#### 2.5.3 Covariates

Covariates were age (<35, 35–64, 65+), sex (female, male), income level (<£30,000 per annum, ≥£30,000 per annum), self-rated health status (poor, good, excellent), neuroticism (low, high), symptoms of anxiety (low, high), symptoms of depression (low, high) and frequency of binge drinking (never, rarely, frequently). The age variable was capped at 100 years, with responses >100 coded as missing (this deviated from the pre-specified analysis plan, in which we had not selected an upper age limit). Self-rated health status was measured by asking: “In general, would you say your health is…” Response options were: 1) poor; 2) fair; 3) good; 4) very good; 5) excellent. For ease of interpretation, response options were collapsed into ‘poor’ (1–2), ‘good’ (3) and ‘excellent’ (4–5). Neuroticism was measured by the Big Five Inventory (John, Donahue, & Kentle, 1991) and symptoms of anxiety and depression were measured by the Goldberg Anxiety and Depression Scales (Goldberg, Bridges, & Grayson, 1988). Responses were transformed into a percentage of maximum possible (POMP) score ranging from 0 to 100. For ease of interpretation, responses were dichotomised using the median split into ‘low’ and ‘high’ neuroticism, anxiety and depression, respectively. Frequency of binge drinking was assessed by the following item: “During the past 30 days, on how many days did you have 5 or more drinks of alcohol in a row, that is, within a couple of hours?” Response options were: 1) 0 days, 2) 1 or 2 days, 3) 3 to 5 days, 4) 6 to 9 days, 5) 10 to 19 days, 6) 20 to 29 days and 7) all 30 days. For ease of interpretation, response options were collapsed into ‘never’ (1), ‘rarely’ (2–3) and ‘frequently’ (4–7). This broadly corresponds to the third item on the validated Alcohol Use Disorders Identification Test-Consumption scale (Frank et al., 2008).

### 2.6 Data analysis

Data were analysed in R v.3.5.2. Respondents with missing data on any of the variables of interest were excluded from the analyses. Descriptive statistics were calculated.

The association of smoking status with bodily pain was assessed in a univariable linear regression analysis with never daily smokers, former daily smokers and daily smokers as the three levels of the explanatory variable and bodily pain as the outcome variable. This was followed by a multivariable linear regression analysis, including the two-way interaction between smoking status and age, adjusting for all covariates. As the two-way interaction was significant, the multivariable regression analysis was repeated in each of the three age groups (<35, 36–64, 65+), omitting age as a covariate. We present the results from the stratified analyses. In a planned sensitivity analysis, the univariable and multivariable analyses were repeated in a series of logistic regression analyses with the dichotomised pain variable as the outcome (i.e. no pain vs. some pain).

In a planned sensitivity analysis, the association of cigarettes per day and bodily pain in daily smokers was assessed in a univariable linear regression analysis. This was followed by a multivariable linear regression analysis, including the two-way interaction between cigarettes per day and age, adjusting for all covariates. As the two-way interaction was significant, the multivariable regression analysis was repeated in each of the three age groups (<35, 36–64, 65+), omitting age as a covariate. We present the results from the stratified analyses in Supplementary File 1.

#### 2.6.1 Power analysis

An *a priori* power analysis using G*Power (Faul, Erdfelder, Lang, & Buchner, 2007) indicated that 921 respondents would provide 99% power to detect a small effect of smoking status on bodily pain (Cohen’s f^2^ = 0.02), with alpha set to 5%.

#### 2.6.2 Bayes Factors

Planned further analyses involved the calculation of Bayes Factors using an online calculator (http://www.lifesci.sussex.ac.uk/home/Zoltan_Dienes/inference/Bayes.htm) to examine whether non-significant associations could best be characterised as evidence of no effect or whether data were insensitive to detect an effect. Prior research has observed a mean difference of −5.76 (95% CI = −1.52 to −10.01) in pain scores between ex- and never-smokers (Lyons, Lo, & Littlepage, 1994). The expected effect size was therefore set to 5.76. The alternative hypothesis was conservatively represented by a half-normal distribution. Bayes Factors (BFs) ≥ 3 can be interpreted as substantial evidence for the alternative hypothesis (and against the null), while BFs of ≤1/3 can be interpreted as evidence for the null hypothesis. BFs between 1/3 and 3 suggest that the data are insensitive to distinguish the alternative hypothesis from the null (Dienes, 2011).

## 3 Results

A total of 588,014 respondents completed the survey between 2009 and 2013, of whom 458,817 (78.0%) resided in the UK and were aged 16 + years. Of these eligible respondents, 31,710 (6.9%) had missing data on sex. Of these, a further 107,514 (25.2%) respondents had missing data on income level. Of the 319,593 respondents with complete data on the sociodemographic characteristics, 25,466 (8.0%) respondents had missing data on bodily pain, self-rated health status and the frequency of binge drinking, 9384 (2.9%) respondents had missing data on neuroticism, symptoms of anxiety and symptoms of depression, and 96,055 (30.1%) respondents had missing data on the second item used to derive the smoking status variable (i.e. “During the past 30 days, on average how many cigarettes did you smoke per day?”), yielding a total of 223,537 respondents with complete data on all variables of interest. The majority of respondents were a never daily smoker (80.4%), with 9.2% former daily smokers and 10.5% daily smokers (see Table 1). The mean level of bodily pain in the total sample was 15.8 (SD = 20.0).

**Table 1:** Smoking, demographic and psychological characteristics of the sample (*N* = 223,537).

	**% (n)**
**Smoking status**	
Never daily smoker	80.4% (179,613)
Former daily smoker	9.2% (20,486)
Daily smoker	10.5% (23,438)
**Age**	
16–34	49.6% (110,920)
35–64	48.2% (107,761)
65+	2.2% (4,856)
**Sex**	
Female	64.4% (144,049)
Male	35.6% (79,488)
**Income**	
<£30,000	50.8% (113,579)
£30,000+	49.2% (109,958)
**Self-rated health status**	
Poor	15.2% (33,924)
Good	27.8% (62,064)
Excellent	57.1% (127,549)
**Neuroticism**	
Low	49.7% (111,184)
High	50.3% (112,353)
**Symptoms of anxiety**	
Low	47.9% (107,074)
High	52.1% (116,463)
**Symptoms of depression**	
Low	47.0% (104,994)
High	53.0% (118,543)
**Frequency of binge drinking**	
Never	56.0% (125,139)
Rarely	22.2% (49,713)
Frequently	21.8% (48,685)
**Cigarettes per day (among current smokers)**	
<1	9.4% (2,204)
1	3.6% (8 4 2)
2–5	20.0% (4,687)
6–10	26.0% (6,101)
11–20	33.1% (7,753)
>20	7.9% (1,851)
**Bodily pain (continuous), M (*SD*)**	
All	15.8 (*20.0*)
Never daily smoker	15.2 (*19.5*)
Former daily smoker	17.5 (*21.1*)
Daily smoker	18.5 (*21.9*)
**Bodily pain (dichotomous), % (n)**	
None	35.6% (79,539)
Some	64.4% (143,998)

Table 2 shows the results from the univariable and multivariable linear regressions predicting bodily pain from smoking status, stratified by age. Former daily and daily smokers reported higher levels of bodily pain than never daily smokers in each age group. In adjusted analyses, being a former daily or daily smoker was associated with a small but significant increase in bodily pain compared with never daily smokers in 16-34-year-olds (*p’s* < 0.01). Being a former daily smoker was associated with a small but significant increase in bodily pain compared with never daily smokers in 35-64-year-olds (*p* < .001) and those aged 65 + years (*p* < .05). The difference between daily and never daily smokers was non-significant in 35-64-year-olds and those aged 65+ years.

**Table 2:** Univariable and multivariable linear regression analyses of the association of smoking status with bodily pain, stratified by age.

	16–34 years (*n* = 110,920)	35–64 years (*n* = 107,761)	65 + years (*n* = 4,856)
	**B (95% CI)**	**B (95% CI)**	**B (95% CI)**
**Smoking status**			
Never daily smoker	ref	ref	ref
Former daily smoker	1.34 (0.89, 1.80)***	1.69 (1.30, 2.07)***	3.75 (1.90, 5.61)***
Daily smoker	3.40 (3.07, 3.73)***	3.76 (3.32, 4.21)***	6.35 (2.63, 10.08)***
	**B_adj_ (95% CI)**	**B_adj_ (95% CI)**	**B_adj_ (95% CI)**
**Smoking status**			
Never daily smoker	ref	ref	ref
Former daily smoker	0.72 (0.30, 1.15)***	1.04 (0.69, 1.38)***	1.65 (0.07, 3.24)*
Daily smoker	0.50 (0.18, 0.82)**	0.09 (−0.31, 0.50)	1.84 (−1.34, 5.02)
**Sex**			
Female	ref	ref	ref
Male	−1.83 (−2.05, −1.61)***	−1.84 (−2.08, −1.59)***	−4.27 (−5.43, −3.10)***
**Income**			
<£30,000	ref	ref	ref
£30,000+	−0.55 (−0.76, −0.34)***	−1.80 (−2.04, −1.57)***	−0.71 (−1.93, 0.51)
**Self-rated health status**			
Poor	ref	ref	ref
Good	−11.35 (−11.68, −11.02)***	−16.48 (−16.83, −16.13)***	−18.49 (−20.18, −16.79)***
Excellent	−17.56 (−17.87, −17.24)***	−25.88 (−26.21, −25.55)***	−31.28 (–32.82, −29.73)***
**Neuroticism**			
Low	ref	ref	ref
High	1.43 (1.07, 1.78)***	1.24 (0.85, 1.63)***	0.49 (−1.42, 2.40)
**Symptoms of anxiety**			
Low	ref	ref	ref
High	−0.37 (−0.68, −0.05)*	−0.69 (−1.03, −0.36)***	−0.60 (−2.18, 0.99)
**Symptoms of depression**			
Low	ref	ref	ref
High	2.10 (1.85, 2.36)***	1.12 (0.84, 1.41)***	1.89 (0.43, 3.36)*
**Frequency of binge drinking**			
Never	ref	ref	ref
Rarely	−1.22 (-1.46, −0.98)***	−1.31 (-1.61, −0.99)***	2.00 (-0.16, 4.15)
Frequently	−1.48 (-1.73, −1.22)***	−1.82 (-2.12, −1.51)***	1.00 (-1.13, 3.13)

*Note.* * *p* < .05; ** *p* < .01; *** *p* < .001.

The calculation of BFs indicated that the data on the association between being a daily smoker and bodily pain in those aged 35–64 years (BF = 0.00) provided evidence for the null hypothesis compared with a large association of B = 5.76. The data on the association between being a daily smoker and bodily pain in those aged 65+ years (BF = 0.58) marginally favoured the null hypothesis compared with a large association but were insensitive to detect an effect.

Table 3 shows results from the planned sensitivity analysis. Former daily and daily smokers had increased odds of reporting some pain than never daily smokers. In adjusted analyses, compared with never daily smokers, former daily smokers had increased odds (*p* < .001) and daily smokers had reduced odds (*p* < .01) of reporting some pain.

**Table 3:** Unadjusted and adjusted Odds Ratios (ORs) for the association between smoking status and the dichotomised pain variable (no pain vs. some pain).

	OR (95% CI)	OR_adj_ (95% CI)
**Smoking status**		
Never daily smoker	1.00	1.00
Former daily smoker	1.13 (1.10, 1.17)***	1.04 (1.02, 1.08)***
Daily smoker	1.18 (1.14, 1.21)***	0.94 (0.91, 0.97)**
**Age**		
16–34		1.00
35–64		1.18 (1.16, 1.21)***
65+		1.84 (1.72, 1.97)***
**Sex**		
Female		1.00
Male		0.87 (0.85, 0.89)***
**Income**		
<£30,000		1.00
£30,000+		0.94 (0.92, 0.96)***
**Self-reported health status**		
Poor		1.00
Good		0.55 (0.53, 0.57)***
Excellent		0.26 (0.25, 0.27)***
**Neuroticism**		
Low		1.00
High		1.15 (1.12, 1.19)***
**Symptoms of anxiety**		
Low		1.00
High		1.04 (1.01, 1.07)**
**Symptoms of depression**		
Low		1.00
High		1.25 (1.22, 1.28)***
**Frequency of binge drinking**		
Never		1.00
Rarely		0.98 (0.95, 1.00)*
Frequently		0.96 (0.94, 0.98)***

*Note.* * *p* < .05; ** *p* < .01; *** *p* < .001.

## 4 Discussion

### 4.1 Principal findings

This study found that former daily and daily smokers in the UK reported higher levels of bodily pain than never daily smokers in each age group. After adjusting for a range of covariates, reported levels of bodily pain in former daily smokers were higher than in never daily smokers at all ages. In respondents aged 16–34 years, daily smokers reported higher pain levels than never daily smokers. In respondents aged 35–64 years and 65+ years, the associations in daily smokers did not reach statistical significance. The calculation of Bayes Factors indicated that the data provided evidence of no difference in those aged 35–64 years, but that they were insensitive to distinguish the alternative hypothesis from the null in those aged 65+ years. In a sensitivity analysis with bodily pain dichotomised into ‘no pain’ and ‘some pain’, former daily smokers had increased, and daily smokers had reduced, odds of reporting some pain compared with never daily smokers. However, due to potential collinearity between the outcome variable and self-reported health status, and increased odds among daily smokers of reporting some pain in the univariable analysis, the results from the multivariable analysis should be interpreted with caution.

The finding that former daily smokers reported higher levels of pain compared with never daily smokers at all ages is consistent with previous research (Bastian et al., 2015, Jakobsson, 2008, John et al., 2006). This raises the possibility that a period of smoking may affect pain experiences, independent of self-rated health status (which at least partly captures smoking-related disease). A potential explanation for the finding that daily smokers aged 16–34 years, but not those aged 35+ years, reported higher levels of pain than never daily smokers is that younger smokers have not yet developed smoking-related disease or general ill health and hence, any smoking-attributable effects on bodily pain are easier to detect. In those aged 35+ years, however, bodily pain may be secondary to specific smoking-related disease or general ill health and hence, smoking-attributable effects on bodily pain must be larger in order to be detected. Indeed, effect sizes in the present study were small, with daily smokers aged 16–34 years experiencing a half-point increase in bodily pain (on a 100-point scale) compared with never daily smokers. However, as age may not serve as an adequate proxy for length or intensity of smoking or time since quitting smoking in former daily smokers, our results should be interpreted with caution and future population surveys should attempt to capture these variables more precisely (e.g. through recording pack-years of smoking).

### 4.2 Strengths and limitations

The BBC Lab UK Study (British Broadcasting Corporation, 2014) provided a useful data source to address the question of whether smoking may result in bodily pain. Due to its large sample size, the BBC Lab UK Study provided sufficient statistical power in subsamples stratified by age. Another key strength of this study was the ability to adjust for a wider range of covariates than previously accounted for, such as self-rated health status, personality (i.e. neuroticism), symptoms of anxiety and depression and alcohol consumption. Ditre and colleagues noted that few studies in the extant literature have controlled for comorbid medical and psychiatric conditions, which may contribute to reports of bodily pain above and beyond smoking status (Ditre, Brandon, Zale, & Meagher, 2011). However, we were unable to adjust for specific smoking-related disease, length of smoking or time since quitting smoking in former daily and daily smokers; it is plausible that long-term, regular smoking (as opposed to any duration of daily smoking during the life course) is associated with higher pain levels. Moreover, as the smoking status variable was derived from two items asking respondents about ever being a daily smoker (as opposed to ever being an occasional smoker), the ‘never daily smoker’ category may have included previous occasional smokers or current very low rate smokers (i.e. occasional smokers who indicated that they had not smoked a single cigarette in the past 30 days). As occasional (i.e. non-daily, very light) smokers are rare in the UK – only 2% in a representative sample of smokers in England (Kotz, Fidler, & West, 2012) – this is unlikely to have substantially affected our results. If occasional smoking were to have an effect on pain, we should have underestimated (rather than overestimated) any association between daily vs. never and non-daily smoking with pain. In addition, the ‘former daily smoker’ category did not capture longer-term cessation as the item used to derive this variable only asked about abstinence for at least 30 days. However, in the nationally representative Smoking Toolkit Study (www.smokinginengland.info), among ex-smokers who have been quit for >1 year and past-year smokers who have been quit for at least 30 days, only 1.4% report having made their most recent quit attempt within the past 3 months, suggesting that the vast majority of those categorised as ex-smokers in the BBC Lab UK Study should have been quit for 3 months or longer.

This study was also limited by employing a cross-sectional study design; there is a paucity of prospective studies examining the relationship of smoking and pain over time (although see Mikkonen et al., 2008 for a prospective study). Smoking rates tend to be higher in those who suffer from chronic pain compared with the general population (Ditre et al., 2011), yet few studies have examined whether chronic pain is prospectively associated with smoking initiation; this merits further investigation. As is commonly the case in smoking research, this study was likely subject to the ‘healthy survivor effect’, a selection process whereby healthy smokers are likely to be over-represented in the older age strata, as those who experience smoking-related problems may stop or die prematurely. This was partly accounted for by stratifying the analyses by age; however, future research should specifically assess the relationship of smoking and pain in a larger sample of older respondents.

Although the sample in the BBC Lab UK Study has previously been found to be representative of the UK population with regards to local authority districts, age and ethnicity (Rentfrow, Jokela, & Lamb, 2015), it is subject to self-selection bias. The proportion of current smokers (10.5%) was substantially lower than the proportions reported in well-established household surveys, such as the Office for National Statistics (Office for National Statistics, 2014) and the English Smoking Toolkit Study (Fidler et al., 2011), in which smoking rates of 19.0% and 19.3% were reported in 2013, respectively. The large number of missingness reduced the dataset from ~500,000 to ~220,000 respondents, which may have introduced further selection bias. Given that the focus in the present study was on within-sample associations (as opposed to prevalence estimates), the lack of representativeness was less of an issue as there was variance in the variables of interest sufficient to detect associations that were present in the population. However, future research should assess whether findings from this study replicate in representative samples of smokers. Respondents aged 65+ years were relatively scarce in this sample. It is hence plausible that respondents aged 65+ years who opted in to respond to the survey are not representative of older adults in the UK. Moreover, the BBC Lab UK Study did not include validated measures of nicotine dependence or patterns of alcohol consumption, such as the Heaviness of Smoking Index (Borland, Yong, O’Connor, Hyland, & Thompson, 2010) or the Alcohol Use Disorders Identification Test (Babor, Higgins-Biddle, Saunders, & Monteiro, 2001). This may limit any comparisons of the results with those in the extant literature. It should also be noted that data were collected between 2009 and 2013; however, we deem it unlikely that the association of smoking and pain has evolved over time.

### 4.3 Implications

On the basis of previous studies observing a positive relationship of smoking status and pain, it has been recommended that smoking cessation and chronic pain services should be integrated (Ditre et al., 2011). Due to the small effect sizes observed in the present study (which may partly be due to the low smoking rates in this non-chronic pain sample), this may not be warranted. However, providers of smoking cessation support in the UK (e.g. the National Centre for Smoking Cessation Training) and elsewhere should consider incorporating materials on coping with pain, particularly targeting younger smokers.

In conclusion, former daily and daily smokers reported higher levels of bodily pain than never daily smokers in each age group. After adjusting for a wide range of characteristics, including health status, neuroticism, anxiety and depression, former daily smokers reported higher levels of bodily pain compared with never daily smokers at all ages. This raises the possibility that a period of smoking at any time during the lifespan results in increased pain.

## Declaration of Competing Interest

OP and CG have no conflicts of interest to declare. LS has received honoraria for talks, an unrestricted research grant and travel expenses to attend meetings and workshops from Pfizer and has acted as paid reviewer for grant awarding bodies and as a paid consultant for healthcare companies. JB has received unrestricted research funding from Pfizer, who manufacture smoking cessation medications. RW undertakes research and consultancy for and receives travel funds and hospitality from manufacturers of smoking cessation medications (Pfizer, GlaxoSmithKline, Johnson & Johnson).

## References

[b0005] Babor T.F., Higgins-Biddle J.C., Saunders J.B., Monteiro M.G. (2001). The alcohol use disorders identification test: Guidelines for use in primary care.

[b0010] Bastian L.A., Fish L.J., Gierisch J.M., Stechuchak K.M., Grambow S.C., Keefe F.J. (2015). Impact of smoking cessation on subsequent pain intensity among chronically ill veterans enrolled in a smoking cessation trial. Journal of Pain and Symptom Manage.

[b0015] Behrend C., Prasarn M., Coyne E., Horodyski M., Wright J., Rechtine G.R. (2012). Smoking cessation related to improved patient-reported pain scores following spinal care. Journal of Bone Joint Surgery.

[b0020] Bendayan R., Cooper R., Muthuri S.G. (2018). Lifetime cigarette smoking and chronic widespread and regional pain in later adulthood: Evidence from the 1946 British birth cohort study. BMJ Open.

[b0025] Bernhard D., Wick G., Wang X., Scott D. (2006). In vitro models for the analysis of cigarette smoke effects. Molecular mechanisms of tobacco-induced diseases.

[b0030] Borland R., Yong H.H., O’Connor R.J., Hyland A., Thompson M.E. (2010). The reliability and predictive validity of the heaviness of smoking index and its two components: findings from the International Tobacco Control Four Country Study. Nicotine & Tobacco Research.

[b0035] Brennan P.L., Schutte K.K., Moos R.H., Brennan P., Park M., Brennan P.L. (2005). Pain and use of alcohol to manage pain: Prevalence and 3-year outcomes among older problem and non-problem drinkers. Addiction.

[b0040] British Broadcasting Corporation (2014). BBC Lab UK. Available from: https://web.archive.org/web/20140918021810/http:/www.bbc.co.uk/labuk/articles/.

[b0045] De Vita M.J., Maisto S.A., Ansell E.B., Zale E.L., Ditre J.W. (2019). Pack-years of tobacco cigarette smoking as a predictor of spontaneous pain reporting and experimental pain reactivity. Experimental and Clinical Psychopharmacology.

[b0050] Dersh J., Polatin P.B., Gatchel R.J. (2002). Chronic pain and psychopathology: research findings and theoretical considerations. Psychosomatic Medicine.

[b0055] Dienes Z. (2011). Bayesian versus orthodox statistics: Which side are you on?. Perspectives on Psychological Science.

[b0060] Ditre J.W., Brandon T.H., Zale E.L., Meagher M.M. (2011). Pain, nicotine, and smoking: research findings and mechanistic considerations. Psychological Bulletin.

[b0065] Faul F., Erdfelder E., Lang A.-G., Buchner A. (2007). G*Power 3: A flexible statistical power analysis program for the social, behavioral, and biomedical sciences. Behavior Research Methods.

[b0070] Fidler J.A., Shahab L., West O., Jarvis M.J., McEwen A., Stapleton J.A. (2011). The smoking toolkit study: A national study of smoking and smoking cessation in England. BMC Public Health.

[b0075] Frank D., DeBenedetti A.F., Volk R.J., Williams E.C., Kivlahan D.R., Bradley K.A. (2008). Effectiveness of the AUDIT-C as a screening test for alcohol misuse in three race/ethnic groups. Journal of General Internal Medicine.

[b0080] Goldberg D., Bridges K., Grayson D. (1988). Detecting anxiety and depression in general medical settings. British Medical Journal.

[b0085] Goldberg D.S., McGee S.J. (2011). Pain as a global public health priority. BMC Public Health.

[b0090] Jakobsson U. (2008). Tobacco use in relation to chronic pain: results from a Swedish population survey. Pain Medicine.

[b0095] Jakobsson U., Larsson C. (2014). Smoking and chronic pain among people aged 65 years and older. Pain Practice.

[b0100] James M.F.M., Duthie A.M., Duffy B.L., McKeag A.M., Rice C.P. (1978). Analgesic effect of ethyl alcohol. British Journal of Anaesthesia.

[b0105] Jenkinson C., Coulter A., Wright L. (1993). Short Form 36 (SF36) health survey questionnaire: normative data for adults of working age. British Medical Journal.

[b0110] John O.P., Donahue E.M., Kentle R.L. (1991). The Big Five Inventory (Versions 4a and 54).

[b0115] John U., Hanke M., Meyer C., Völzke H., Baumeister S.E., Alte D. (2006). Tobacco smoking in relation to pain in a national general population survey. Preventive Medicine.

[b0120] Kotz D., Fidler J., West R. (2012). Very low rate and light smokers: Smoking patterns and cessation-related behaviour in England, 2006–11. Addiction.

[b0125] Lyons R.A., Lo S.V., Littlepage B.N. (1994). Perception of health amongst ever-smokers and never-smokers: A comparison using the SF-36 Health Survey Questionnaire. Tobacco Control.

[b0130] Mikkonen P., Leino-Arjas P., Remes J., Zitting P., Taimela S., Karppinen J. (2008). Is smoking a risk factor for low back pain in adolescents? A prospective cohort study. Spine.

[b0135] Munafò M.R., Zetteler J.I., Clark T.G. (2007). Personality and smoking status: A meta-analysis. Nicotine & Tobacco Research.

[b0140] Office for National Statistics (2014). Adult smoking habits in Great Britain, 2013. Available from: https://www.ons.gov.uk/peoplepopulationandcommunity/healthandsocialcare/healthandlifeexpectancies/compendium/opinionsandlifestylesurvey/2015-03-19/adultsmokinghabitsingreatbritain2013.

[b0145] Palmer K.T., Syddall H., Cooper C., Coggon D. (2003). Smoking and musculoskeletal disorders: Findings from a British national survey. Annals of the Rheumatic Diseases.

[b0150] Parkerson H.A., Zvolensky M.J., Asmundson G.J.G. (2013). Understanding the relationship between smoking and pain. Expert Review of Neurotherapeutics.

[b0155] Petre B., Torbey S., Griffith J.W., De Oliveira G., Hermann K., Mansour A. (2015). Smoking increases risk of pain chronification through shared corticostriatal circuitry. Human Brain Mapping.

[b0160] Phillips C.J. (2009). The cost and burden of chronic pain. Reviews of Pain.

[b0165] Radwanski M. (1992). Self-medicating practices for managing chronic pain after spinal cord injury. Rehabilitation Nursing.

[b0170] Rentfrow P.J., Jokela M., Lamb M.E. (2015). Regional Personality Differences in Great Britain. PLoS ONE.

[b0175] Shi Y., Weingarten T.N., Mantilla C.B., Hooten M.W., Warner D.O. (2010). Smoking and pain: pathophysiology and clinical implications. Anesthesiology.

[b0180] Von Elm E., Altman D.G., Egger M., Pocock S.J., Gøtzsche C., Vandenbroucke J.P. (2007). The strengthening the reporting of observational studies in epidemiology (STROBE) statement: Guidelines for reporting observational studies. Lancet.

[b0185] Williams A.C., Craig K.D. (2016). Updating the definition of pain. Pain.

[b0190] World Health Organization (2017). WHO report on the global tobacco epidemic, 2017: Monitoring tobacco use and prevention policies. World Health Organization. https://apps.who.int/iris/handle/10665/255874.

[b0195] Zale E.L., Maisto S.A., Ditre J.W. (2016). Anxiety and depression in bidirectional relations between pain and smoking: implications for smoking cessation. Behavior Modification.

