# Supplementary material for: Associations between smoking status and bodily pain in a cross-sectional survey of UK respondents
Source: Addict Behav. 2020 Mar;102:106229. doi: 10.1016/j.addbeh.2019.106229 (PMC6959457; doi:10.1016/j.addbeh.2019.106229)
Supplement: Supplementary data 1 [file mmc1.docx]

*Table.* Univariable and multivariable linear regression analyses predicting bodily pain from cigarettes per day in daily smokers, stratified by age.

|  | **16-34 years (n = 13,817)** |  | **35-64 years (n = 9,470)** |  | **65+ years (n = 151)** |
| --- | --- | --- | --- | --- | --- |
|  | **B (95% CI)** |  | **B (95% CI)** |  | **B (95% CI)** |
| **Cigarettes per day** |  |  |  |  |  |
| <1 | ref |  | ref |  | ref |
| 1 | 0.27 (-1.67, 2.20) |  | -1.19 (-4.59, 2.21) |  | 33.85 (5.63, 62.07)* |
| 2-5 | 1.88 (0.65, 3.12)** |  | 1.26 (-0.90, 3.43) |  | 9.89 (-10.87, 30.66) |
| 6-10 | 2.33 (1.12, 3.53)*** |  | 2.63 (0.59, 4.67)* |  | 10.94 (-7.28, 29.15) |
| 11-20 | 4.08 (2.88, 5.28)*** |  | 4.56 (2.62, 6.49)*** |  | 4.29 (-13.44, 22.03) |
| >20 | 11.10 (9.21, 12.99)*** |  | 8.70 (6.48, 10.92)*** |  | 21.97 (2.80, 41.14)* |
|  | **B_adj_ (95% CI)** |  | **B_adj_ (95% CI)** |  | **B_adj_ (95% CI)** |
| **Cigarettes per day** |  |  |  |  |  |
| <1 | ref |  | ref |  | ref |
| 1 | -0.01 (-1.84, 1.81) |  | -0.88 (-3.90, 2.15) |  | 14.81 (-7.99, 37.61) |
| 2-5 | 0.78 (-0.39, 1.95) |  | 0.05 (-1.88, 1.97) |  | 1.97 (-14.10, 18.03) |
| 6-10 | 0.24 (-0.90, 1.39) |  | 0.11 (-1.71, 1.93) |  | -7.72 (-22.42, 6.98) |
| 11-20 | 1.02 (-0.13, 2.17) |  | 0.38 (-1.35, 2.11) |  | -3.74 (-17.76, 10.27) |
| >20 | 5.56 (3.75, 7.38)*** |  | 1.26 (-0.74, 3.26) |  | -1.11 (-11.86, 2.33) |
| **Sex** |  |  |  |  |  |
| Female | ref |  | ref |  | ref |
| Male | -2.10 (-2.79, -1.41)*** |  | -2.06 (-2.97, -1.16)*** |  | -4.77 (-11.86, 2.33) |
| **Income** |  |  |  |  |  |
| <£30,000 | ref |  | ref |  | ref |
| £30,000+ | -1.05 (-1.78, -0.31)** |  | -2.50 (-3.37, -1.64)*** |  | 6.81 (-1.04, 14.67) |
| **Self-reported health status** |  |  |  |  |  |
| Poor | ref |  | ref |  | ref |
| Good | -10.56 (-11.40, -9.72)*** |  | -17.14 (-18.25, -16.03)*** |  | -27.39 (-36.78, -18.00)*** |
| Excellent | -15.35 (-16.22, -14.48)*** |  | -25.28 (-26.40, -24.15)*** |  | -42.43 (-50.94, -33.93)*** |
| **Neuroticism** |  |  |  |  |  |
| Low | ref |  | ref |  | ref |
| High | 2.05 (0.92, 3.18)*** |  | 2.23 (0.80, 3.66)** |  | -9.92 (-21.18, 1.35) |
| **Symptoms of anxiety** |  |  |  |  |  |
| Low | ref |  | ref |  | ref |
| High | -0.72 (-1.72, 0.27) |  | -0.83 (-2.07, 0.40) |  | 10.42 (0.50, 20.34)* |
| **Symptoms of depression** |  |  |  |  |  |
| Low | ref |  | ref |  | ref |
| High | 2.69 (1.85, 3.53)*** |  | 0.61 (-0.46, 1.68) |  | 7.27 (-0.67, 15.21) |
| **Frequency of binge drinking** |  |  |  |  |  |
| Never | ref |  | ref |  | ref |
| Rarely | -1.39 (-2.25, -0.53)** |  | -2.65 (-3.76, -1.54)*** |  | -3.33 (-15.22, 8.55) |
| Frequently | -2.11 (-2.90, -1.33)*** |  | -3.88 (-4.86, -2.90)*** |  | -2.82 (-13.58, 7.93) |

*Note.* * *p* < .05; ** *p* < .01; *** *p* < .001.
